# Case report: Successful medical management of adrenocortical carcinoma with metastasis in a Maltese dog

**DOI:** 10.3389/fvets.2023.1142418

**Published:** 2023-07-14

**Authors:** Sin-Wook Park, Keon Kim, Ock-Kyu Kim, Woong-Bin Ro, Chang-Min Lee

**Affiliations:** ^1^Department of Veterinary Internal Medicine, College of Veterinary Medicine and BK21 FOUR Program for Creative Veterinary Science Research Center, Chonnam National University, Gwangju, South Korea; ^2^Cat Vet Animal Hospital, Seongnam-si, Gyeonggi-do, South Korea

**Keywords:** adrenal-dependent hyperadrenocorticism, canine, adrenal tumor, metastasis, trilostane

## Abstract

**Introduction:**

Adrenocortical carcinoma (ACC) with metastasis has a grave prognosis, and adrenalectomy is associated with a high perioperative mortality rate in dogs. A favorable outcome following trilostane treatment in patients with metastatic ACC confirmed by a decreased size of the adrenal tumor and metastatic lesions has not been reported in dogs.

**Case description:**

A 12-year-old neutered male Maltese dog was diagnosed with a right adrenal tumor and a hepatic mass. Adrenal-dependent hyperadrenocorticism (ADH) was diagnosed based on clinical signs and an adrenocorticotropic hormone stimulation test (ACTHST). In addition, tests for plasma metanephrine and normetanephrine ruled out a pheochromocytoma. Based on cytology and computed tomography, unresectable metastatic ACC was confirmed. The dog was managed with trilostane due to the presence of distant metastasis. Medical management improved the clinical signs and post-ACTHST cortisol concentrations. One year after the first presentation, the clinical signs and ACTHST test showed a favorable outcome. In addition, computed tomography revealed a decreased size of the right adrenal tumor and resolution of the hepatic mass.

**Conclusions:**

Trilostane could be considered as a treatment option for unresectable metastatic ACC. A decrease in tumor size following treatment with trilostane has not been reported in dogs. This case report is the first to demonstrate a favorable outcome of metastatic ACC following trilostane mono therapy for >1 year.

## Introduction

Primary adrenal tumors account for 0.17 to 0.76% of all neoplasia in dogs (1, 2). Adrenal-dependent hyperadrenocorticism (ADH) is uncommon, and is the cause of Cushing's syndrome in approximately 15% of dogs with this syndrome (1, 2). Tumors originating from the adrenal cortex are generally adrenocortical carcinomas (ACCs) or adenoma and may produce steroid hormones such as androgens, aldosterone, and cortisol (3). One study reported that approximately 50% of dogs with ACC have local invasion of the mass into the caudal vena cava (CVC) (1). Metastatic rates of 7–50% have been reported in dogs with ACC, and may include the liver, lungs, kidneys, and mesenteric lymph nodes (1, 3).

Treatment for adrenal tumors may include medical and/or surgical procedures. Treatment methods are often recommended based on whether local invasion or distant metastasis is present. Medical treatment is often favored when the primary tumor appears unresectable (2). However, due to high surgical treatment complication rates (15–50%) and perioperative mortality rates (5–29%) associated with adrenalectomy, only medical treatment is performed in some dogs with resectable adrenal tumors (2, 4–6). In dogs, medical treatment for adrenal tumors that secrete cortisol includes mitotane and trilostane (4).

Trilostane selectively inhibits the enzyme 3β-hydroxysteroid dehydrogenase (HSD3B1) corticoids and sex hormones by blocking the conversion of pregnenolone to progesterone (7). A recent study reported that the survival times of dogs with ADH treated with trilostane or mitotane were not significantly different, and that adverse effects were more frequently observed in dogs receiving mitotane (4).

To the authors' knowledge, there are no previous reports of successful management of a metastatic adrenocortical tumor with trilostane monotherapy. This case report is the first to demonstrate a favorable outcome of metastatic ACC following trilostane mono therapy for >1 year.

## Case description

**Figure 1:**
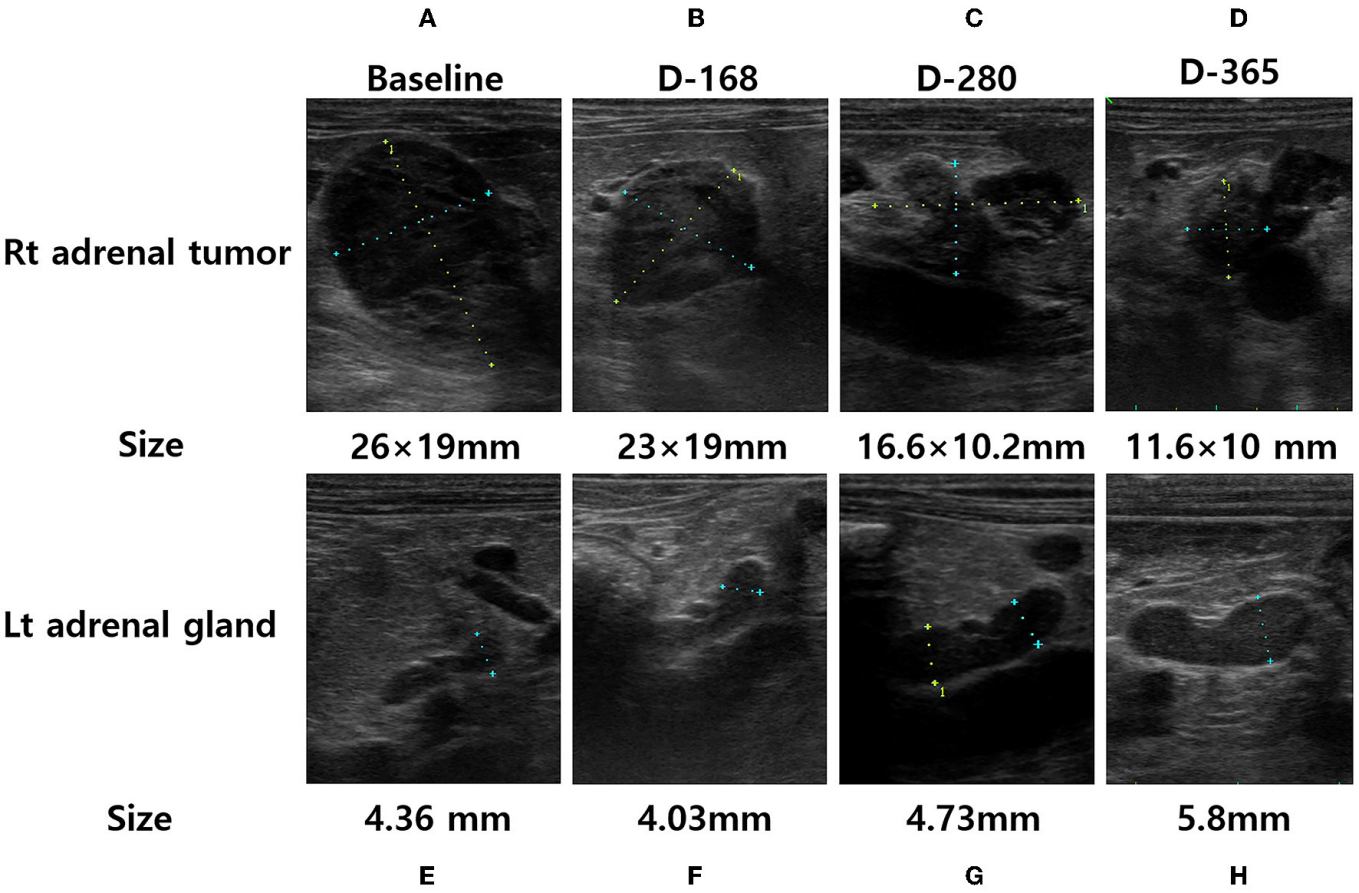
Adrenal ultrasonography of a dog performed at the initial presentation [**(A)**, right adrenal tumor and **(E)**, left adrenal gland], after 168 days of trilostane treatment [**(B)**, right adrenal tumor and **(F)**, left adrenal gland], after 280 days of trilostane treatment [**(C)**, right adrenal tumor and **(G)**, left adrenal gland], and after one year of trilostane treatment [**(D)**, right adrenal tumor and **(H)**, left adrenal gland]. The size of the right adrenal tumor was decreased within one year [**(A, D)**, 26 × 19mm → 11.6 × 10mm]. The size of the cranial pole of left adrenal gland was slightly increased within 1 year [**(E, H)**, 4.36mm → 5.8mm].

A 12-year-old, neutered male, Maltese dog was presented to a local animal hospital with abdominal distension, polyuria, and polydipsia. The abdominal mass was confirmed on ultrasonography, and the patient was referred to our veterinary medical teaching hospital for additional diagnostics and management of the abdominal mass. On physical examination, generalized erythema, scales, alopecia and lichenification were observed. In addition, inguinal hyperpigmentation and abdominal dissension was noted. No abnormal body temperature, heart rate, and respiratory rate was observed. A complete blood count, serum biochemical analysis, and electrolyte and coagulation tests were performed, and elevated alkaline phosphatase (517 U/L; reference range, 23–212 U/L), triglycerides (>375 mg/dL; reference range, 10–100 mg/dL), and lactate (2.9 mmol/L; reference range, 0.5–2.5 mmol/L) were revealed. On urinalysis, proteinuria (2+, 100 mg/dL) and hematuria (2+, 50 μL) were identified. Radiography showed hepatomegaly and calculi in the urinary bladder. Abdominal ultrasonography showed gallbladder sludge, one heterogenous hepatic mass (15 × 5 mm), and a right adrenal tumor (26 × 19 mm) with local invasion of the CVC; the left adrenal gland was within normal limits (Figure 1).

**Figure 2:**
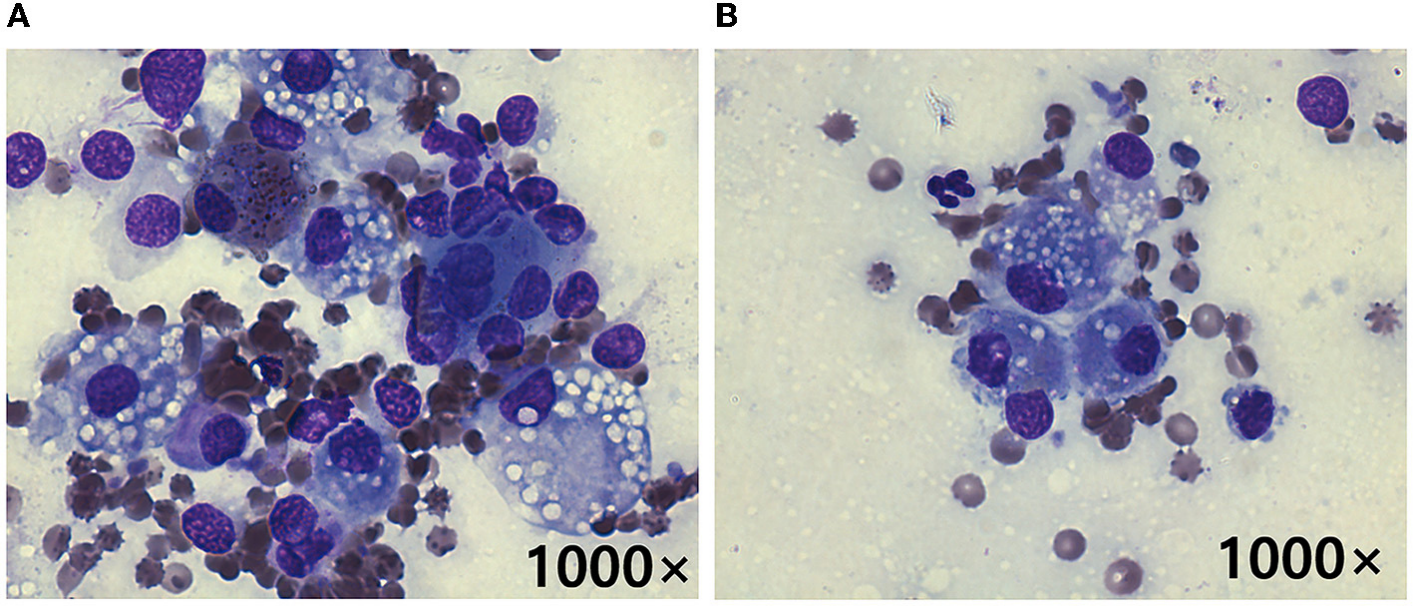
Cytology following fine-needle aspiration of the right adrenal tumor **(A)** and hepatic mass **(B)** in a dog with metastatic adrenocortical carcinoma. Cells with anisocytosis, a basophilic cytoplasm, and cytoplasmic vacuolization suspected to be originating from the adrenal cortex are seen in both **(A, B)**. These findings indicate that the hepatic mass likely represented a metastatic ACC (Diff-Quik stain, original magnification 1000×).

Fine-needle aspiration cytology performed by a clinical pathologist on the adrenal tumor and hepatic mass revealed cells with anisocytosis, round nuclei, a basophilic cytoplasm, and cytoplasmic vacuolization; these were suspected to have originated from the adrenal cortex (Figure 2). In addition, hepatocytes with cytoplasmic rarefaction (indistinct vacuolation) were observed that might occurs secondary to hyperadrenocorticism. An adrenocorticotropic hormone (ACTH; Synacthen; Dalim Bio tech, Korea) stimulation test (5 μg/kg IV) showed elevated cortisol concentrations (pre-stimulation, 6.1 μg/dL; reference range, 2–6 μg/dL; post-stimulation, 29.4 μg/dL; reference range, 6–18 μg/dL). The plasma concentrations of metanephrine (0.78 nmol/L; reference range, 0.4–1.7 nmol/L) and normetanephrine (3.67; reference range, 1.5–6.6 nmol/L) were within the normal ranges. The plasma ACTH concentration was below the reference range (< 1.2 pg/ml; reference range, 10–100 pg/ml). Based on these results, hyperadrenocorticism (HAC) caused by right ACC was diagnosed.

**Figure 3:**
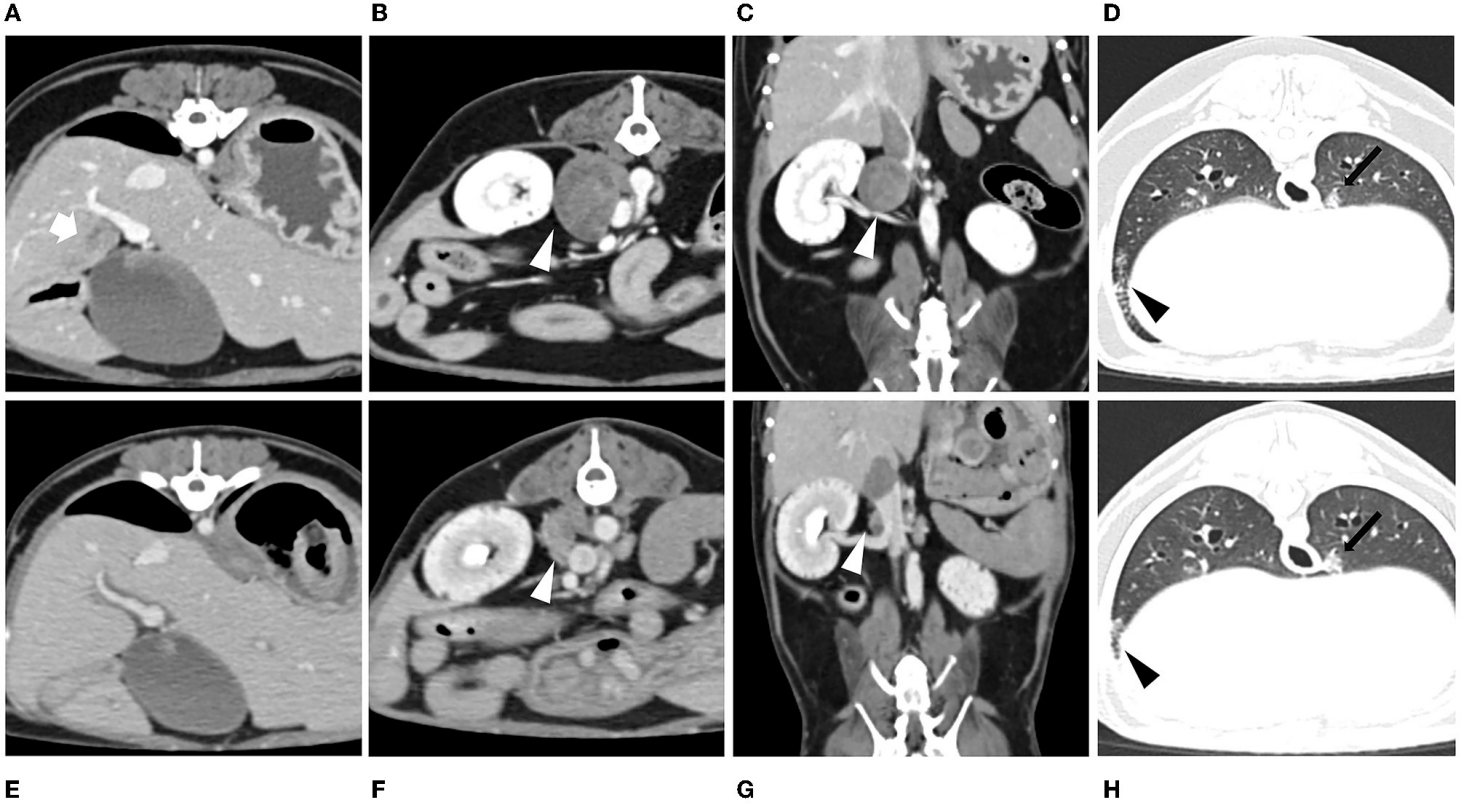
Post-contrast CT images obtained at the time of initial presentation [**(A, B, D)** transverse view; **(C)** dorsal view] and after 1 year of trilostane treatment [**(E, F, H)** transverse view; **(G)** dorsal view]. During therapy, the diameter of the adrenal tumor decreased [**(B, C, F, G)** white arrowhead]. The metastatic liver mass with heterogenous enhancement [**(A)** white arrow] resolved after trilostane treatment **(E)**. No significant changes of ground-glass opacities [GGOs; **(D, H)** black arrowhead] and lung masses [**(D, H)** black arrow] were found after treatment.

To evaluate for metastasis and determine the surgical options for the tumor, computed tomography (CT) was performed by radiologist using Siemens SOMATOM Emotion eco (16-slice CT scanner) with following scan parameters: 120mAs, 130kVp and 1-mm continuous slices. The contrast of total-body CT scanning was obtained after administration of iohexol (Omnipaque 300 mg iodine/ml, 3 mg/kg, intravenous bolus injection). A CT scan showed a right adrenal tumor (width × height × length, 17 × 21 × 22 mm^3^) with CVC invasion, a hepatic mass (width × height × length, 13 × 9 × 22 mm^3^), and >20 soft tissue attenuating solid masses (longest diameter, 3–6 mm) and ground-glass opacities (GGOs) throughout the lungs, indicating suspected metastasis from ACC (Figure 3). Based on the clinical signs, hormone analysis, CT findings, and cytologic results, the dog was diagnosed with ACC with metastasis that was considered to be unresectable. Radiation therapy was discussed with the owner but ultimately declined. Therefore, the adrenal tumor was managed with trilostane (1 mg/kg orally q12h). On day 28, the results of an ACTHST 4-h post administration of trilostane remained above the reference range (post-stimulation, 19.8 μg/dL; reference range, 1.45–9.1 μg/dL), and the clinical signs did not improve; therefore, the dosage of trilostane was doubled from the initial dosage (2 mg/kg orally q12h). On day 56, the patient's clinical signs and the results of an ACTHST were within the reference range (post-stimulation, 6.2 μg/dL; reference range, 1.45–9.1 μg/dL), showing a favorable outcome. The regular rechecks showed good clinical control based on the patient's clinical signs and the results of ACTHST, hematology, and biochemistry and the treatment was continued for 11 months (Table 1). During the treatment period, while the size of the adrenal tumor decreased, there was a tendency for the size of the contralateral adrenal gland to increase (Figure 1).

**Table 1:** ACTHST, hematology, and biochemistry results at various days after diagnosis and during treatment.

	**Day 0**	**Day 28**	**Day 56**	**Day 112**	**Day 168**	**Day 224**	**Day 280**	**Day 336**	**Day 365**	**Reference range**
Pre-ACTH	**6.1**	**9.1**	1.8	3.3	7.2	3.0	2.6	2.2	4	1.6–5 (μg/dL)
Post-ACTH	**29.4**	**19.8**	6.2	**10.6**	**13.3**	**9.5**	**5.2**	6.8	8.6	5.4–9.1 (μg/dL)
White blood cell count	10.48	11.42	12.12		13.56		9.45	10.58	10.75	5.05–16.76 (10^9^/L)
Neutrophils	8.46	9.39	10.10		**11.78**		7.55	8.85	8.55	2.95–11.64 (K/μL)
Lymphocytes	1.20	1.25	1.40		1.13		1.14	**0.93**	1.15	1.05–5.10 (K/μL)
Monocytes	0.44	0.42	0.36		0.36		0.48	0.54	0.37	0.16–1.12 (K/μL)
Eosinophils	0.36	0.35	0.25		0.27		0.27	0.24	0.64	0.06–1.23 (K/μL)
Basophils	0.02	0.01	0.01		0.02		0.01	0.02	0.04	0.00–0.10 (K/μL)
Red blood cells	7.08	6.79	7.23		6.37		6.56	6.16	6.49	5.65–8.87 (M/μL)
Hematocrit	47.9	45.4	49.3		42.7		43.8	41.2	42.5	37.3–61.7 (%)
Sodium	147.4	145.7	143.1		144.7		144.2	146.5	146.5	140–150 (mmol/L)
Potassium	4.40	4.23	3.77		3.79		4.33	3.96	3.87	3.5–5.8 (mmol/L)
Chloride	111.2	113.7	117.6		114.6		115	115.3	115	109–120 (mmol/L)
Phosphate	3.2	3.8	4.2				4.0	4.1	3.9	2.5–6.8 (mg/dL)
Calcium	10.3	10.9	10.8				10.5	10.3	10	7.9–12.0 (mg/dL)
Glucose	109	116	120				118	125	108	74–143 (mg/dL)
Albumin	3.0	3.4	3.2				3.2	3.3	3.4	2.3–4.0 (g/dL)
Globulin	**4.7**	4.2	4.2				4.4	4.3	4.2	2.5–4.5 (g/dL)
Alkaline phosphatase	**517**	**445**	**382**		400		297	204	159	23–212 (U/L)
Alanine aminotransferase	60	47	67				30	27	34	10–100 (U/L)
Urea	10	19	**6**				14	12	15	7–27 (mg/dL)
Creatinine	**0.4**	0.6	0.5				0.6	0.6	0.7	0.5–1.8 (mg/dL)
Triglycerides	**>375**	**>375**	**>375**		**>375**		**>375**	**295**	**159**	10–100 (mg/dL)

Abnormal results are presented in bold.

One year after the initial presentation, the patient's clinical signs and lab work showed a favorable outcome. In addition, abdominal ultrasonography and CT was performed to re-stage metastasis of the adrenal tumor. Ultrasonography revealed a decreased size of right adrenal tumor (11.6 × 10 mm) and an increased size of the left adrenal gland (5.8 mm). The CT scan revealed the right adrenal gland had decreased (width × height × length, 12 × 15 × 11 mm^3^), the left adrenal gland increased (width × height × length, 7.4 × 5.9 × 18 mm^3^), the hepatic mass resolved completely, and no significant changes occurred in number or appearance of the masses and GGOs in the lungs. In addition, no additional metastasis was observed (Figure 3). A few months later, the patient underwent surgery for bladder stones and cholecystectomy for gall bladder sludge at the local hospital and died shortly thereafter due to gallbladder rupture. A necropsy was not performed.

## Discussion

The dog in this report showed the typical clinical signs, serum chemistry abnormalities, and response to specific endocrinological tests for HAC (8). This dog was suspect to have metastatic ACC based on CT imaging and cytologic examination of the adrenal tumor and hepatic mass. In addition, since there are reports that adrenal functional tumors may secrete medullary hormones as well as adrenocortical hormones, pheochromocytoma was excluded based on metanephrine and normetanephrine concentrations related to catecholamines (9). Trilostane therapy for HAC in dogs with adrenocortical metastasis has been reported to be safe and acceptable for palliative treatment (10). Likewise, the subsequent reduction in polyphagia, polyuria, and regrowth of hair would be consistent with successful trilostane therapy in this case. Moreover, the decreased size of the right adrenal tumor and resolution of the hepatic mass were confirmed by CT. A reduction in tumor size induced by a treatment with no known antimitotic effects has not been reported in dogs. Thus, the present case may be meaningful for clinicians in that trilostane showed a favorable outcome in a patient with metastatic ACC.

ACC is the most common adrenal tumor in dogs, followed by pheochromocytoma, adenoma, and aldosteronoma (2, 11). ACC may produce excessive adrenal hormones such as androgens, aldosterone, and cortisol (2). On diagnostic imaging, an adrenal tumor >20 mm in diameter may indicate malignant ACC or pheochromocytoma in dogs (11–13). In addition, local invasion of the CVC and distant organ metastasis may be useful indicators of malignancy. However, ultrasonographic features are not specific for the type of primary adrenal neoplasia (11). Histopathology would be required to demonstrate the tumor type and to determine the best treatment option, but the owner would not allow invasive procedures in this case. Therefore, treatment was performed based on the clinical signs, diagnostic imaging findings, and hormonal analyses.

In human medicine, adrenalectomy is the primary treatment option for adrenal tumors even when surgical resection is incomplete. When incomplete resection occurs, treatment with mitotane is indicated, and if it is not effective, systemic chemotherapy is performed (14). Likewise, surgical resection has been performed in dogs with adrenal tumors in veterinary clinics (15–17). However, a high perioperative mortality rate of adrenalectomy has been reported in dogs (5–29%) (2, 4, 5). Therefore, medical management has been recommended as an alternative treatment, and a recent study revealed that the choice of medical treatment (mitotane or trilostane) does not affect survival time in dogs with ADH (4). In addition, recent studies investigating the management of adrenal tumors with trilostane in dogs with unresectable adrenal tumors showed favorable outcome (6, 10).

In the present case, the reduction in the size of the adrenal tumor and resolution of the hepatic mass were confirmed by diagnostic imaging (ultrasonography and CT). Mitotane significantly decreases the adrenal gland size in dogs with pituitary-dependent hyperadrenocorticism (PDH), and it also is effective in dogs with ADH due to its adrenocorticolysis effect (4). In contrast, there are reports that trilostane treatment may cause ultrasonographically detectable enlargement of the adrenal glands in dogs with both PDH and ADH (10, 18–20). A possible explanation for this is that hyperplasia of the adrenal cortex may reflect increased synthesis of precursors due to increased secretion of ACTH, which is the result of abolishing the negative feedback by cortisol (19). However, there are a few reports that trilostane may cause adrenal cortical necrosis that leads to decreased size of the bilateral adrenal glands in dogs with PDH (20–22). Therefore, we suspect the decreased size of the adrenal tumor may have been the result of necrosis of the adrenal cortex; however, confirmatory histopathology was not performed.

There are reports that the contralateral adrenal gland may shrink in dogs with ADH, but there was no significant evidence of adrenal cortical atrophy in this case, and the adrenal gland was similar to those of normal dogs based on the ultrasonographic features of size, shape, and parenchymal structure (11, 23). During trilostane treatment in this patient, the size of the contralateral adrenal gland was increased, which might have been the result of adrenal cortical hyperplasia (19). In contrast, the sizes of the adrenal tumor significantly decreased, and the metastatic hepatic mass was resolved. The reason for these findings is unknown, but it is suspected that trilostane may have had an antimitotic effect on the adrenal tumor.

Trilostane has been used to treat cancers (e. g., breast and hepatocellular carcinoma) in human medicine. (24–27). One study showed that the HSD3B1 gene is involved in the growth of hepatocellular carcinoma, and trilostane significantly inhibited the growth of hepatocellular carcinoma by inhibiting the HSD3B1 gene (24). In addition, since HSD3B1 expression has been associated with a poor prognosis in patients with breast cancer, treatment with trilostane may yield favorable outcomes by blocking cell proliferation in those patients who express the HSD3B1 gene (26, 28). Although the mechanism of action of trilostane remains unclear, trilostane may be recommended as an option in dogs with unresectable ADH because it may suppress the growth of ACC, as shown in this case.

The definitive evidence for the effectiveness of a treatment for a tumor is the improvement in clinical signs and survival. However, images are generally used to assess therapeutic effects earlier and more objectively (29). Current response assessments are based on changes in tumor size measured by CT or other diagnostic imaging (30). According to the canine response evaluation criteria in solid tumors (RECIST v 1.0), the dog in this report achieved a partial response following treatment with trilostane (29). Evaluating tumor size (CT is the preferred modality for assessment of lesions) as well as monitoring clinical signs and hormone analyses are important when prescribing trilostane to dogs with ADH to monitor response to treatment and better determine prognosis.

This is the first case report of trilostane treatment in a dog with metastatic ACC with a confirmed favorable outcome. The low incidence of side effects of trilostane suggests that trilostane is a highly acceptable alternative to mitotane for medical management of ADH. Studies with a larger number of dogs are necessary to confirm the value of trilostane treatment for ACC with metastasis.

## Data availability statement

The original contributions presented in the study are included in the article/supplementary material, further inquiries can be directed to the corresponding authors.

## Ethics statement

Since this is a case report, ethical review and approval were not required. Written informed consent was obtained from the owners for the participation of their animals in this study.

## Author contributions

S-WP and KK were involved in case analysis and were responsible for writing the manuscript. O-KK was involved in the draft preparation and case analysis. W-BR and C-ML were involved in the coordination of the case and were responsible for interpretation of results. All authors contributed to the article and approved the submitted version.
